# A SARS-CoV-2 Outbreak Illustrating the Challenges in Limiting the Spread of the Virus — Hopi Tribe, May–June 2020

**DOI:** 10.15585/mmwr.mm6944a5

**Published:** 2020-11-06

**Authors:** Jocelyn Hirschman, Harpriya Kaur, Kay Honanie, Royce Jenkins, Duane A. Humeyestewa, Rachel M. Burke, Tracy M. Billy, Oren Mayer, Mose Herne, Mark Anderson, Ravikiran Bhairavabhotla, Graydon Yatabe, S. Arunmozhi Balajee

**Affiliations:** ^1^Hopi Health Care Center, Polacca, Arizona; ^2^CDC COVID-19 Response Team; ^3^Hopi Tribe, Kykotsmovi, Arizona; ^4^Laboratory Leadership Service, CDC

On June 3, 2020, a woman aged 73 years (patient A) with symptoms consistent with coronavirus disease 2019 (COVID-19) (*1*) was evaluated at the emergency department of the Hopi Health Care Center (HHCC, an Indian Health Services facility) and received a positive test result for SARS-CoV-2, the virus that causes COVID-19. The patient’s symptoms commenced on May 27, and a sibling (patient B) of the patient experienced symptom onset the following day. On May 23, both patients had driven together and spent time in a retail store in Flagstaff, Arizona. Because of their similar exposures, symptom onset dates, and overlapping close contacts, these patients are referred to as co-index patients. The co-index patients had a total of 58 primary (i.e., direct) and secondary contacts (i.e., contacts of a primary contact); among these, 27 (47%) received positive SARS-CoV-2 test results. Four (15%) of the 27 contacts who became ill were household members of co-index patient B, 14 (52%) had attended family gatherings, one was a child who might have transmitted SARS-CoV-2 to six contacts, and eight (30%) were community members. Findings from the outbreak investigation prompted the HHCC and Hopi Tribe leadership to strengthen community education through community health representatives, public health nurses, and radio campaigns. In communities with similar extended family interaction, emphasizing safe ways to stay in touch, along with wearing a mask, frequent hand washing, and physical distancing might help limit the spread of disease.

The Hopi are a Native American tribe and a sovereign nation, primarily residing on the 1.5 million-acre Hopi Reservation in northeastern Arizona (*2*). The Hopi Tribe leadership declared a COVID-19–associated state of emergency on March 17, which was followed by a stay-at-home order March 23 (with projected reopening on June 20). On April 13, HHCC reported its first laboratory-confirmed COVID-19 case in a patient residing on the Hopi Reservation, and cases continued to be diagnosed at low levels through May. However, at the beginning of June, HHCC reported that during the preceding 14 days, the number of new cases had increased sharply, from 1–2 to 10–15 per day (J Hirschman, MD, CDC, personal communication, June 2020).

## Investigation and Results

On June 3, a woman aged 73 years (patient A) with symptoms consistent with COVID-19 (*1*) was seen at HHCC emergency department and received a positive SARS-CoV-2 RNA amplification rapid diagnostic test (Abbott ID NOW) (*3*) result. On June 4, HHCC’s Community Health Department began contact tracing. Patient A was interviewed to identify symptom onset date and any persons with whom she had close contact* from 2 days before symptom onset on May 27 until the interview date. Contact tracing interviews revealed that a sibling of patient A, aged 67 years, (patient B) experienced symptoms on May 28, 1 day after patient A’s symptom onset. Patients A and B reported spending a few hours on May 23, in a large home improvement store in Flagstaff, while wearing masks and reported intermittent mask-wearing^†^ in the 2-hour drive home, and no other passengers were in the car. On May 27, 4 days after returning from Flagstaff, patient A reported headache and continued feeling unwell during the following 4 days (May 28–31). During June 1–2, she experienced worsening headache with fever and shortness of breath and received a positive SARS-CoV-2 test result on June 3. Patient B reported runny nose and sore throat on May 28 and received a positive SARS-CoV-2 test result on June 5. Both patients A and B self-isolated after receiving positive test results, and are considered co-index patients because of their similar exposures, symptom onset dates, and overlapping close contacts.

Primary contacts of co-index patients A and B with laboratory-confirmed COVID-19 test results and secondary contacts (contacts of primary contacts) (*4*) were interviewed by telephone and asked to come to HHCC for testing. Interviews were conducted using a standardized form.^§^ Close contacts who received positive test results were classified as symptomatic if their symptoms were consistent with COVID-19 (*1*), presymptomatic if they were asymptomatic when tested but experienced symptoms within 14 days of exposure, or asymptomatic if they never reported any symptoms. These contacts were not retested after they became symptomatic. Symptomatic contacts were tested with Abbott ID NOW and received same-day results; symptomatic contacts with negative test results were retested using real-time reverse transcription–polymerase chain reaction (RT-PCR) (*5*). All contacts who were asymptomatic at the time of investigation were tested with RT-PCR.

**FIGURE 1:**
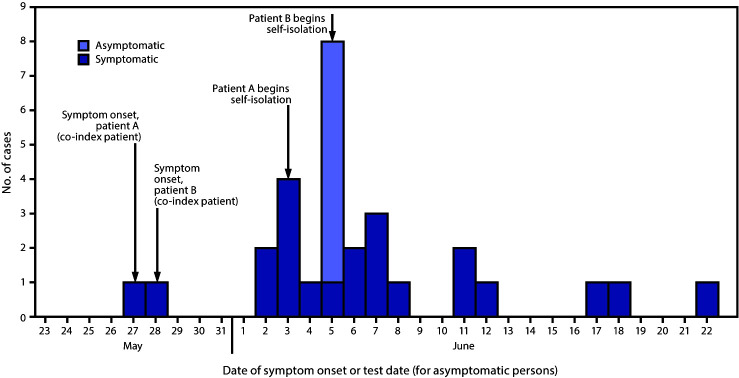
Date of symptom onset or test date (for asymptomatic persons) in a cluster of 29 laboratory-confirmed COVID-19 patients — Hopi Tribe, May–June 2020 **Abbreviation:** COVID-19 = coronavirus disease 2019.

Overall, 58 primary and secondary contacts of index patients A and B were identified, 27 (47%) of whom received positive test results for SARS-CoV-2. Among the 29 persons with confirmed COVID-19 (including co-index patients A and B), 22 (76%) were symptomatic and seven were asymptomatic (Figure 1). To describe the sequence of transmission, contacts of patients A and B are denoted by letters and numbers, indicating contact with either or both patients, and the hypothesized sequence of transmission.

**FIGURE 2:**
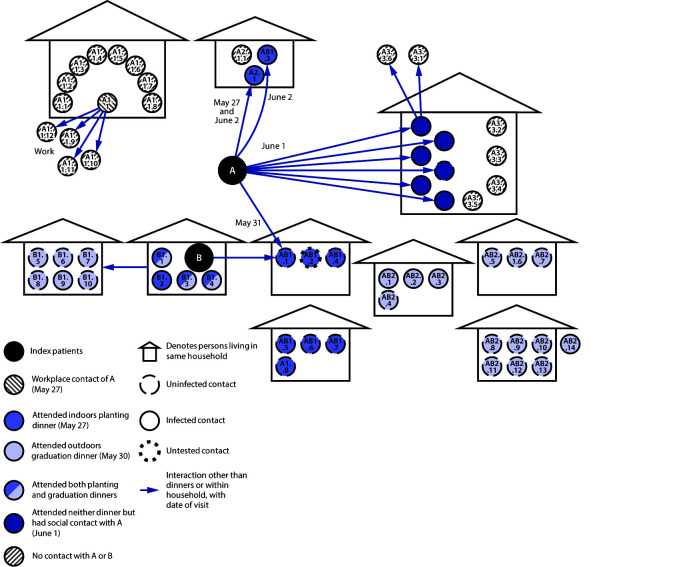
Transmission of SARS-CoV-2 among 58 primary and secondary contacts* of co-index patients A and B, resulting in 27 (47%) confirmed cases — Hopi Tribe, May–June 2020 * Patient AB2.14 lives in another city but is part of the AB2.8–AB2.14 family.

Patient A reported working in an office on May 27, her symptom onset date, in close proximity to a colleague (A1.1) for approximately 6 hours (Figure 2). Both patients A and A1.1 reported intermittent mask-wearing in the enclosed office. On June 2, patient A1.1 experienced diarrhea and loss of appetite and, on June 4, received a positive SARS-CoV-2 test result. While patient A1.1 was symptomatic and before being tested, she had contact with 12 persons, including eight household members (A1.1.1–A1.1.8) and four colleagues (A1.1.9–A1.1.12). All 12 contacts of patient A1.1 were tested for SARS-CoV-2, and seven household contacts (all but A1.1.8) received positive test results; all four colleagues received negative test results. After work on May 27, patient A visited a second sibling (patient A2.1).

On the date her symptoms began, May 27, patient A also worked outdoors with patient B and 13 extended family members (A2.1, B1.1–B1.4, and AB1.1–AB1.8^¶^) and then dined with them at an indoor potluck planting dinner,** where attendees did not wear masks. On May 30, patients A and B attended a graduation dinner with 23 persons in addition to themselves (B1.1, B1.3–B1.10, and AB2.1–AB2.14). Three persons who attended the planting dinner also attended the graduation dinner (B1.1, B1.3, and B1.4). These three persons, five persons who attended only the graduation dinner (AB2.1, AB2.2, AB2.3, AB2.14, and B1.9), and three who attended only the planting dinner (A2.1, AB1.3, and B1.2) later developed symptoms and received positive SARS-CoV-2 test results. Both dinners took place during the stay-at-home order. On May 31, patients A and B had contact with another family member (AB1.1), for approximately 1 hour, although AB1.1 took a telephone call outside most of that time. AB1.1 received a negative SARS-CoV-2 test result and remained asymptomatic.

On June 1, 4 days after patient A’s symptom onset, she was visited for several hours by her daughter (A3.1), son-in-law (A3.2), and four grandchildren (A3.3–A3.6). Later that day, one grandchild (A3.3) played with two friends. On June 2, patient A3.3 developed fever, cough, chills, and difficulty breathing. She was taken to HHCC on June 3 and received a positive SARS-CoV-2 test result. On June 4, all nine household members of A3.3 (A3.1, A3.2, A3.4–A3.6, and A3.3.2–3.3.5) were tested for SARS-CoV-2, and two received positive results, including patient A3.4, who had visited patient A, and patient A3.3.2, who had not. That same day, one of the granddaughter’s playmates (A3.3.1) also received a positive test result. No other infections were detected in the playmate’s household,^††^ and everyone in this household reported consistent mask-wearing, including at home, after patient A3.3.1’s symptom onset. Patients A3.4 and A3.3.2 became symptomatic on June 6 and 7, respectively. On June 8, patient A3.1 became symptomatic and received a positive test result on June 10. On June 11, patients A3.2 and A3.5 became symptomatic and received positive test results.

On June 2, patient A’s sibling (A2.1) and nephew (AB1.3) visited her for a few hours. They experienced symptoms on June 7 and received positive SARS-CoV-2 test results on June 9. On June 22, A2.1’s spouse (A2.1.1) received a positive test result after experiencing symptoms.

Patient B lives with four family members, and six extended family members live next door. From May 28 (symptom onset date) through June 5 (date of laboratory confirmation), patient B had close contact with these 10 family members (B1.1–B1.10), including five (four household and one next door) who later became symptomatic and were confirmed to have COVID-19.

Overall, among 60 extended family and community member contacts, including co-index patients A and B, 29 (48%) persons with confirmed COVID-19 were identified. The median patient age was 21 years (range = 1–79 years) (Table). Among patients with confirmed COVID-19, 13 (45%) had at least one underlying medical condition, including seven (24%) with obesity, three (10%) with diabetes, and three (10%) with cardiovascular disease. Four patients (14%) were presymptomatic, all of whom were aged <20 years, and seven (24%) were asymptomatic, six of whom were aged <30 years. Among 27 contacts with confirmed COVID-19, seven (24%) had visited patient A, four (14%) were household members of patient B, one (4%) was a workplace contact, and one was a child who had six close contacts who received positive test results. Two patients went to an emergency department, one patient required critical care, and no deaths occurred. Contact tracing interviews revealed limited understanding of how and when to wear masks, adhere to physical distancing of ≥6 feet, and practice hand hygiene.

**TABLE:** Demographic and clinical characteristics of persons with laboratory-confirmed SARS-CoV-2 infection (N = 29) — Hopi Tribe, May–June 2020

Characteristic	No. (%)
**Median age (range)**	21 (1–79)
**Age group (yrs)**	
0–19	13 (45)
20–39	8 (28)
40–59	4 (14)
60–79	4 (14)
**Sex**	
Male	11 (38)
Female	18 (62)
**Chronic underlying conditions**	
Cardiovascular diseases	3 (10)
Chronic lung disease	2 (7)
Chronic renal disease	1 (3)
Diabetes mellitus	3 (10)
Hyperglycemia	2 (7)
Obesity	7 (24)
Other*	4 (14)
**Signs and symptoms**	
Abdominal pain	4 (14)
Asymptomatic	7 (24)
Chills	6 (21)
Cough	10 (35)
Diarrhea	5 (17)
Difficulty breathing	4 (14)
Fever	11 (38)
Headache	9 (31)
Malaise	1 (3)
Muscle ache	2 (7)
Runny nose	11 (38)
Sinus congestion	1 (3)
Sore throat	2 (7.0)
**Known setting of primary contact** ^†^	
Graduation reception dinner**^§^**	8 (30)
Household	4 (14)
Household visits	7(24)
Planting dinner	6 (22)
Work	1 (4)
**Laboratory testing results**	
**Total positive** ^¶^	29 (48)
Symptomatic**	22 (76)
Presymptomatic^††^	4 (14)
**Total positive/Total no. tested (attack rate, %)**	**29/60 (48)**

* Other includes high cholesterol, impaired glucose tolerance, depressive disorder, dysuria, nuclear sclerosis, proteinuria, and hyperlipidemia.

^†^ Both numerator and denominator excluded co-index patients.

^§^ Three persons attended both planting dinner and graduation dinner.

^¶^ Includes co-index patients A and B.

** Includes presymptomatic.

^††^ Developed symptoms after receiving positive test results; patients were not retested after they became symptomatic.

## Public Health Response

HHCC led the overall response in collaboration with the Hopi Tribe. The Hopi Emergency Response Team, working in collaboration with HHCC public health nurses, coordinated support for housing, food, and other needs during isolation and quarantine and set up a communication team focused on community education and mitigation with messaging regarding recommended mask-wearing, hand hygiene, and physical distancing.

## Discussion

In this COVID-19 outbreak among the Hopi, two gatherings of extended family members and workplace exposure likely facilitated transmission of SARS-CoV-2 beyond household contacts into the broader community. Both gatherings included >10 persons and took place while the stay-at-home order was active. A distinctive element of Hopi lifestyle highlighted by this investigation was the frequent social interaction among extended family members, leading to repeated exposure of contacts to patients A and B, both of whom were symptomatic ≥1 week before testing, during which time they socialized in the community. Consistent with other reports, many children and young adults with COVID-19 were asymptomatic or had mild symptoms (*6*,*7*); these patients might not have been identified without universal contact testing. One child exposed six contacts who were later confirmed to have COVID-19, including five household members; although four household members had also visited patient A, the intervals between cases as well as interview findings suggest that the child might have introduced COVID-19 into the household. Approximately one half of the COVID-19 patients identified in this outbreak had chronic underlying conditions.

This investigation highlights a need for prevention strategies focused on enhanced community education related to recognition of COVID-19 symptoms and encouraging consistent mask-wearing. All household members, including the index patient, should wear masks within shared spaces in the household. These strategies, along with self-isolation upon symptom onset, can limit exposures and mitigate transmission. After this investigation, HHCC and the Hopi Tribe increased community messaging in English and in Hopi, using multiple modalities including radio and in-person messaging through community health representatives. Messaging explained that by wearing a mask, practicing physical distancing, washing one’s hands frequently, and taking other preventive measures in accordance with CDC guidelines, persons can reduce the risk to themselves and others (*8*,*9*).

The findings in this report are subject to at least three limitations. First, despite intense contact tracing efforts, some tertiary contacts might have been missed. Second, precise determination of exposure dates and classification of contacts as secondary versus tertiary was challenging because of the repeated and overlapping interactions among extended family members. Finally, detailed information on mask wearing and physical distancing practices could not be consistently obtained.

Overall, collaborative efforts and prompt communication between HHCC and the Hopi Tribe proved crucial in containing this outbreak. Lessons learned in this outbreak might also prove useful for other communities with multigenerational households and frequent interactions among extended family members.

**Summary**

**What is already known about this topic?**

Large gatherings pose a risk for SARS-CoV-2 transmission.

**What is added by this report?**

Among 60 immediate and extended family and community members of the Hopi Tribe, 29 (48%) laboratory-confirmed COVID-19 cases occurred; 14% were presymptomatic, and 24% of patients were asymptomatic. The majority of presymptomatic and asymptomatic cases occurred in children and young adults.

**What are the implications for public health practice?**

Frequent and recurring social interactions among extended family members permitted repeated exposures to infectious persons. In communities with similar extended family interaction, emphasizing safe ways to stay in touch, together with wearing a mask, frequent hand washing, and physical distancing might help limit the spread of disease.

## Footnotes

* Close contact is defined as someone who was within 6 feet of an infected person for at least 15 minutes starting from 2 days before illness onset.

† Intermittent mask wearing was defined as tribal member’s acknowledgment of not wearing a mask throughout the duration of close contact.

§ CDC’s Human Infection with 2019 Novel Coronavirus Case Report Form (https://www.cdc.gov/coronavirus/2019-ncov/downloads/pui-form.pdf) was used for case investigations. A customized form was used for contact-tracing interviews.

¶ One person who attended the planting dinner (AB1.2) refused testing and was not included in the study results.

** The planting dinner is an activity common among Hopi Tribe members, in which a family member invites immediate and extended members to work together in the crop fields in the morning and then hosts a dinner for those invited later in the evening.

†† These tertiary contacts are not included in the analysis denominator.
